# High-Temperature Piezoelectric Sensing

**DOI:** 10.3390/s140100144

**Published:** 2013-12-20

**Authors:** Xiaoning Jiang, Kyungrim Kim, Shujun Zhang, Joseph Johnson, Giovanni Salazar

**Affiliations:** 1 Department of Mechanical and Aerospace Engineering, North Carolina State University, Raleigh, NC 27695, USA; E-Mails: kkim8@ncsu.edu (K.K.); jajohns9@ncsu.edu (J.J.); gsalaza@ncsu.edu (G.S.); 2 Materials Research Institute, Pennsylvania State University, University Park, PA 16802, USA; E-Mail: soz1@psu.edu

**Keywords:** high-temperature sensing, high-temperature piezoelectrics, piezoelectric sensors, high-temperature piezoelectric sensors

## Abstract

Piezoelectric sensing is of increasing interest for high-temperature applications in aerospace, automotive, power plants and material processing due to its low cost, compact sensor size and simple signal conditioning, in comparison with other high-temperature sensing techniques. This paper presented an overview of high-temperature piezoelectric sensing techniques. Firstly, different types of high-temperature piezoelectric single crystals, electrode materials, and their pros and cons are discussed. Secondly, recent work on high-temperature piezoelectric sensors including accelerometer, surface acoustic wave sensor, ultrasound transducer, acoustic emission sensor, gas sensor, and pressure sensor for temperatures up to 1,250 °C were reviewed. Finally, discussions of existing challenges and future work for high-temperature piezoelectric sensing are presented.

## 1. Introduction

Sensing technologies for use in ultra-high-temperatures (>800 °C) are in great demand, particularly in the automotive, aerospace, and energy industries. As an example, in an aerospace propulsion system, high-temperature (HT) sensors are necessary for intelligent propulsion system design, operation and for enhancement of system maintenance and safety [1,2]. Specifically, HT sensors are used to monitor propulsion component conditions and the incoming data is analyzed to optimize propulsion system operations under temperatures of 500–1,000 °C, and with lifetimes up to 100,000 h [3]. However, conventional microelectromechanical systems (MEMS) and piezoelectric sensors cannot function at such high temperatures, and thus, these sensor devices must be located in areas with controlled environments [1]. This limitation of non-direct sensing-induced inaccuracy leads to decreased fuel efficiency and reduced reliability. In space exploration technology, high-temperature devices are also essential. For example, the planet Venus is known for its harsh environmental conditions, including high-temperatures (460 °C), high pressure (9 MPa), and corrosiveness [1,4], which has been a challenge to conventional sensors. In automotive combustion systems, HT sensors are essential for recording engine temperature, pressure, and vibration to improve the efficiency and reliability of internal combustion engines [3,5]. Among various sensing applications, combustion sensors or knock sensors are subject to the harshest environments because these sensors need to be located as close as possible to the high-temperature source (e.g., the combustion engine) for accurate monitoring [6]. These sensors are usually required to work properly at temperatures greater than 1,000 °C, and with vibration sweeping up to 10 *g* (*g* = 9.8 m/s^2^) for a relatively long time [5]. Besides, the energy and manufacturing industries consistently demand high-temperature sensing techniques. For example, nuclear power industry adopts ultrasonic transducers at high-temperatures for non-destructive testing (NDT) and nondestructive evaluation (NDE) of various critical components, and hence, obtains the internal state of the materials or structures [4]. In manufacturing plants, ultrasonic NDT of metal components is usually performed at temperatures >400 °C. Therefore, HT sensors are in critical need in a broad range of industries, as well as in new materials development and scientific studies.

## 2. High-Temperature Sensing Techniques

Extensive research has been conducted on piezoresistive, capacitive, fiber optic, and piezoelectric sensors for high-temperature applications [7–9]. Piezoresistive sensors utilize the electrical resistance change in response to external excitations such as force, pressure or acceleration. In general, the piezoresistive sensors are less susceptible to electromagnetic interference (EMI). However, the inherent temperature dependence of material resistivity can lead to inaccuracies for ultra-high-temperature applications [10–12]. Capacitive sensors usually consist of two separated capacitor plates (parallel plate type) or comb drive type of structures. The distance between the plates or the overlapped area of the comb structures changes upon applied stress, and hence leads to capacitance changes. Capacitive sensors have the advantage of low thermal drift, high resolution and good noise performance. However, this type of sensor suffers from limited robustness and is easily influenced by parasitic capacitance with magnitudes similar to that of the sensor itself [13–16]. Fiber optic sensors are also widely used for high-temperature applications because of their immunity to electromagnetic interference and high operating temperatures (<2,000 °C). For example, single-crystal sapphire optical sensors have gained great attention due to their robustness, chemical corrosion resistance, and high melting point (2,040 °C). It was demonstrated that the sapphire optical fiber sensor can be used at temperatures up to 1,100 °C without high-temperature degradations [17]. However, this type of sensor requires special packaging to protect the mechanically fragile fiber wires and fiber tips. This can limit the use of fiber optic sensors at high-temperatures due to a mismatch of the thermal expansion coefficient between packaging and fiber materials which leads to mechanical failures of sensors [18]. Furthermore, a complicated fabrication process and an expensive and complex signal processing system are disadvantages of fiber optic sensors for widespread use [18–20]. Conventional piezoelectric sensing has been applied widely in sensing of vibration, pressure, mass, distance, chemical and bio-sensing, but its operation temperature is usually below 700 °C. Recent development on high-temperature piezoelectric sensing suggests piezoelectric sensing at temperatures close to 1,000 °C. Table 1 summarizes key features for different types of HT sensors.

## 3. HT Piezoelectric (HTPE) Sensing

### 3.1. HT Piezoelectric Crystals

Simple structure, fast response time, and ease of integration, all give high-temperature piezoelectric (HTPE) sensors an advantage and make them of particular interest. Various piezoelectric materials have been extensively researched for high-temperature applications, including quartz (SiO_2_), lithium niobate (LiNbO_3_, LN), gallium orthophosphate (GaPO_4_), langasite (La_3_Ga_5_SiO_14_, LGS) and aluminum nitride (AlN) [21,22]. Each of these materials has its own unique advantages and drawbacks for use in HT sensors, as shown in Table 2. Quartz, the most popular HT piezoelectric material, possesses high electrical resistivity (>10^17^ Ω at room temperature), high mechanical quality factor, and excellent high-temperature stability. Nevertheless, its low electromechanical and piezoelectric coefficients, high losses above 350 °C, and α to β phase transition temperature at 573 °C limit the use of quartz for high-temperature applications [3,23]. The Curie temperature of LiNbO_3_ has been reported to be about 1,150 °C with high electromechanical coefficients. However, practically, this material is limited to 600 °C due to chemical decomposition (starting at 300 °C), increased attenuation and intergrowth transition, loss of oxygen to the environment, and limited resistivity. Lithium niobate also suffers from a short lifetime at elevated temperatures: 10 days at 400 °C and 0.1 days at 450 °C due to its decomposition [24–26]. GaPO_4_ shares some properties with quartz, such as high electrical resistivity and mechanical quality factor, but higher electromechanical coupling and greater piezoelectric sensitivity up to its α to β phase transition (<970 °C). This type of crystal possesses fairly sharp resonances and significantly lower viscosities compared to other high-temperature piezoelectric materials [27]. However, it is limited to operation temperatures below 700 °C because of decreased mechanical quality factor due to increased structural disorder, limiting its usage at high-temperatures [21,22]. Langasite crystals, which have been extensively studied for high-temperature applications including bulk acoustic wave (BAW) and surface acoustic wave (SAW) applications, have no phase transition prior to their melting points (1,470 °C) [28,29]. However, oxygen vacancies transport and diffuse in the lattice limits the sensing performance of langasite sensors, producing low electrical resistivity and low quality factor at elevated temperatures [29–31]. Another popular HT piezoelectric material is aluminum nitride (AlN). AlN is a non-ferroelectric material with melting point being on the order of 2,200 °C. AlN thin film has been reported to be used as a high frequency ultrasonic transducer or a surface acoustic wave sensor, maintaining its piezoelectric properties up to 1,150 °C. Nonetheless, AlN mass production has remained a challenge due to the difficulties in creating high quality and large size bulk piezoelectric materials from AlN [4,32,33]. Rare earth calcium oxyborate single crystals ReCa_4_O(BO_3_)_3_ (ReCOB, Re: rare earth elements such as Gd, La, and Y) have recently gained special attention for ultra-high-temperature applications [34]. In particular, YCa_4_O(BO_3_)_3_ (YCOB) has been shown to be a promising candidate for HT sensors due to its exceptionally high resistivity, stable piezoelectric properties, and lack of phase transformation before the melting temperature of 1,500 °C [35,36], as shown in Table 2. In reported high-temperature research (>1,000 °C), the only limitation of YCOB sensors was the degradation of platinum thin film electrodes.

### 3.2. Electrodes for High-Temperature Piezoelectric Sensing

Thin film electrodes (∼100 nm thickness) are commonly used for piezoelectric devices to apply an electric field or to obtain a generated charge signal. In order to be effectively used in high-temperature sensors, these thin film electrodes should be capable of long term high-temperature operation [40,41]. Various thin film electrodes, including Pt, Pt-based alloys, and other metallic alloys or conductive ceramic electrodes have been researched for high-temperature sensing applications.

Platinum thin film electrodes are popular for both low and high-temperature sensors due to their excellent electrical properties, high melting point and outstanding oxidation resistance [42]. Nevertheless, there are some limitations to its use at temperatures higher than 650 °C because of platinum thin films' degradation phenomena as a result of agglomeration, recrystallization, and dewetting effects [43]. Agglomeration, which is a nucleation and growth process, is one of the most dominant degradation mechanisms of thin metal films at high-temperatures [44]. This phenomenon is related to surface diffusivity which can be rapid for platinum and this leads to malfunction of high-temperature piezoelectric sensor with Pt thin film electrodes due to the loss of thin film's electrical conductivity [45]. It has been reported that Pt-based alloys such as Pt-Rh, Pt-Ir and Pt-Zr can be used without surface degradation for temperatures up to 750 °C [46]. Recently Pt-Rh/ZrO_2_ thin film, Pt/Rh alloy co-deposited with ZrO_2_, has been developed for high-temperature applications, displaying stable and consistent performance up to 850 °C. When protective SiAlON ceramic coatings were applied to the device, the electrode performance was enhanced [40,43,47]. Ir-based electrodes such as Ir/TiAlN have also been studied for HT applications, but their operating temperature was limited up to 700 °C [48,49]. Therefore, bulk or thick film electrodes may be a good option for high-temperature sensors since the use of thin film electrodes mentioned above is limited by the degradation at temperatures above 850 °C. On the other hand, conductive ceramic electrodes such as LSM (La_0.65_Sr_0.35_MnO_3_) and LSCF (La_0.6_Sr_0.4_Co_0.2_Fe_0.8_O_3_) can be good alternatives to metal electrodes due to their good long-term stability at high-temperatures (<800 °C) [44,50–52]. However, typically these materials have disadvantages of the lower conductivity in the low-temperature range compared to the metal electrodes. Table 3 summarizes the important properties of thin film electrodes.

### 3.3. HT PE Sensors

#### 3.3.1. HT PE Accelerometer

A high-temperature accelerometer is widely used for detecting the combustion engines' abnormal behavior such as a knock effect [53–56]. For knock detecting applications, accelerometers should have high operational temperature and high rigidity due to the excessively harsh working conditions of the combustion engine including high pressure (80 bars) and high-temperature (1,227 °C) as well as high *g* environment [53]. The high-temperature piezoelectric accelerometer is one of the most attractive accelerometer types for this application because of its high sensitivity, stability over a broad range of temperatures, high rigidity, simple and cheap manufacturing process.

**Figure 1.**
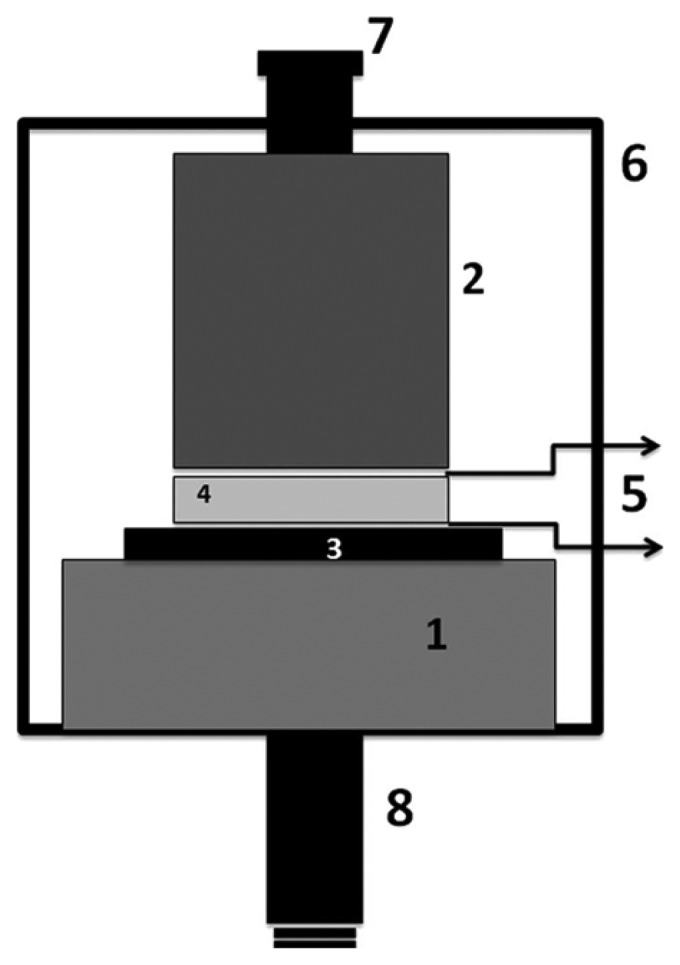
Schematic diagram of the monolithic compression-mode accelerometer assembly [57] (copyright 2010 by the American Institute of Physics).

**Figure 2.**
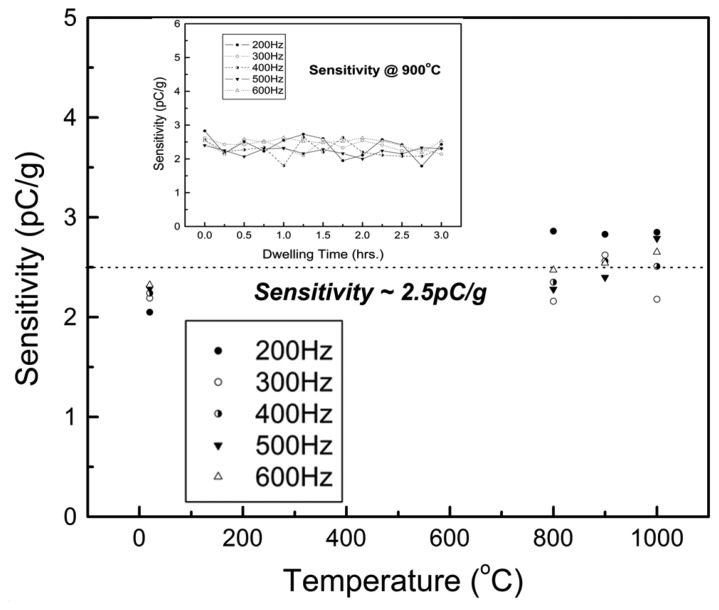
The measured sensitivities of the YCOB accelerometer as a function of temperature up to 1,000 °C with a frequency range of 100–600 Hz. The small inset shows the measured sensitivities as a function of dwell time at 900 °C for 3 h [57] (copyright 2010 by the American Institute of Physics).

Zhang *et al.* have successfully demonstrated a compression mode piezoelectric accelerometer using YCOB single crystals for ultrahigh-temperature applications [57]. Thickness mode YCOB crystals (15 × 7 × 2 mm^2^) with the (*XYlw*)-15°/45° cut were prepared and used for piezoelectric sensing crystals. Inconel and high purity alumina were mainly used for the materials of HT sensor assembly and a platinum foil was used for electrical connection. A schematic diagram of the monolithic compression-mode accelerometer assembly was shown in Figure 1. A screw (7) was used to compress the piezoelectric element (4) between the seismic mass (2) and the base plate (1). A high purity alumina (3) was chosen for electrical insulation and the top and bottom electrodes (5) are made of platinum foil. The accelerometer performance was verified by experimental results as a function of temperature up to 1,000 °C with a frequency range of 100–600 Hz. The stable sensitivity of the prototyped accelerometer was found to be 2.4 ± 0.4 pC/*g* across the measured temperature and frequency range as shown in Figure 2.

The sensitivity as a function of dwell time at 900 °C for 3 h was shown in the small inset in Figure 2 and the sensitivity was found to be 2.4 ± 0.4 pC/*g*. These results suggest the possibility of using YCOB single crystals as PE sensors and acoustic sensors for ultrahigh-temperature applications.

**Figure 3.**
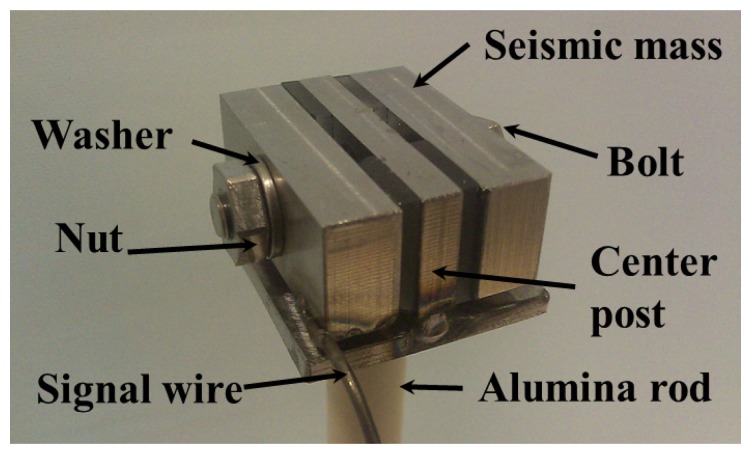
A photograph of the shear mode accelerometer assembly for high-temperature applications [58] (copyright 2012 by the Elsevier).

Recently, Kim *et al.* have investigated a shear mode piezoelectric accelerometer with optimized structure design for high-temperature applications using YCOB single crystals [58]. For high-temperature applications, shear mode sensors can offer higher temperature stability with reduced thermal effects from the sensor base, compared to the compression mode sensor. Figure 3 shows the assembly of the shear mode piezoelectric accelerometer. Platinum electrodes were not used for this sensor in order to avoid sensor failures due to thin film electrode degradation at high-temperatures. In addition, the assembly was accomplished by tightening the nut and bolt to compensate for the thermal expansion effect of each component.

**Figure 4.**
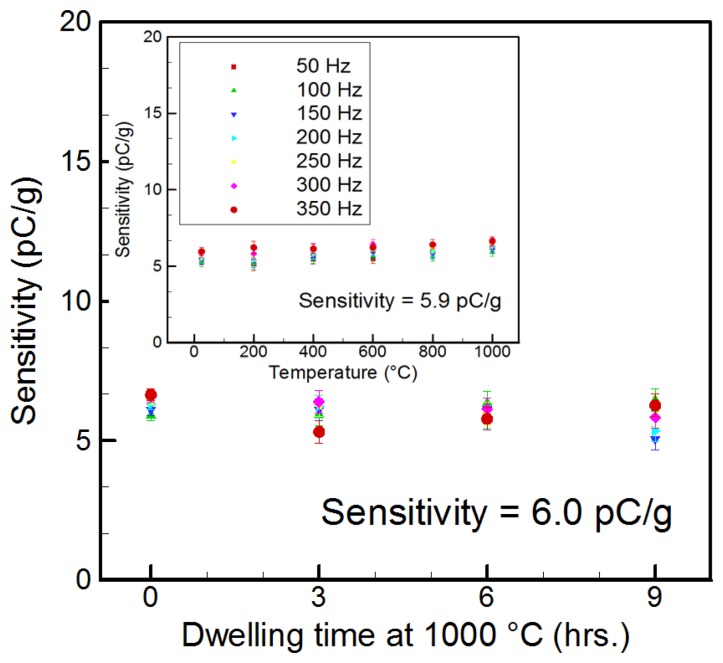
The measured sensitivity of the shear mode accelerometer with increasing temperature (25–1,000 °C) at the tested frequency range (50–350 Hz). The inset presents that the long-term sensitivity for the 9-hour dwell time test [58] (copyright 2012 by the Elsevier).

Figure 4 shows the measured sensitivity of the shear mode accelerometer with increasing temperature (25–1,000 °C) at the tested frequency range (50–350 Hz). The highly stable sensitivity of 5.9 ± 0.06 pC/g was observed throughout the tested frequency and temperature range. From the 9-hour dwell time test, the long-term stable sensitivity (6.0 ± 0.12 pC/*g*) at 1,000 °C was also obtained as can be seen in the inset in Figure 4.

**Figure 5.**
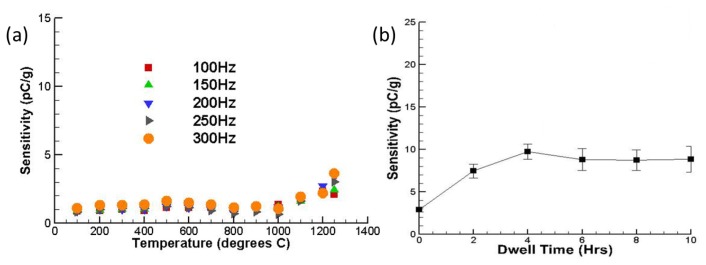
(**a**) The sensor sensitivity at increasing temperatures (25–1,250 °C) for different frequencies (100–300 Hz). (**b**) The measured average sensitivity during a 10-hour dwelling test at 1,250 °C [59] (copyright 2012 by the SPIE).

More recently, further studies of piezoelectric accelerometers using YCOB crystals for higher temperatures (≈1,300 °C) have been conducted by Salazar *et al.* [59]. A similar design including the shear mode type and electrode-less structure was used for the prototyped accelerometer. Accelerometer sensitivities were measured at increasing temperatures up to 1,250 °C. The excellent stability up to 1,000 °C as previously reported for YCOB sensors was obtained. The differences in reported sensitivity come from testing the YCOB crystals and Inconel parts multiple times, affecting both materials' properties. YCOB crystals of different sizes were used for each test as well, which would have a different corresponding capacitance. Figure 5a shows the sensor sensitivity at increasing temperatures with different frequencies. As the temperature was increased beyond 1,000 °C, a gradual and steady increase in sensor sensitivity (≈2.92 ± 0.56 pC/*g* at 1,250 °C) was observed. The sensitivity of the accelerometer was also measured during a 10 h dwell test. After two hours of testing, the sensitivity of the accelerometer reached a quasi-steady state (≈8.7 ± 1.63 pC/*g*), which indicated that the prototyped accelerometer can operate reliably at 1,250 °C as shown in Figure 5b. This increase and plateau in the accelerometer sensitivity with increasing temperature may be due to the seismic masses and center post softening at high-temperatures, allowing for a better contact surface, which would increase charge transfer efficiency.

Many high-temperature piezoelectric accelerometers are commercially available from companies including PCB Piezotronics, Inc. (Depew, NY, USA) and Meggitt Sensing Systems (Dorset, UK) [60,61]. These accelerometers are usually charge mode output accelerometers because that the voltage mode piezoelectric sensors contain built-in signal conditioning electronics which cannot operate at high-temperatures (>163 °C). Charge mode output accelerometers require external signal conditioning equipment, e.g., laboratory style charge amplifier or fixed in-line charge converter. As an example, the sensitivity of PCB Series 357 can vary from 3.5 to 100 pC/*g* and can operate at temperatures up to 649 °C.

#### 3.3.2. HT PE Surface Acoustic Wave Sensor

Surface acoustic wave (SAW) devices such as SAW filters, delay lines and resonators are widely used in consumer electronics including television sets and mobile phones due to their high performance, small size, and good reproducibility [62]. One of the important advantages for SAW-based sensors is the capability of wireless sensing without using cables or additional energy sources [63]. Wireless passive SAW sensors can be preferred for certain applications which require indirect contact or high-temperature measurements. For these reasons, HT wireless SAW sensors can be suitable for monitoring temperatures, pressures or gas concentrations for high-temperature applications such as HT furnaces or process chambers for semiconductors or ceramics manufacturing [24]. For example, the real-time temperature measurement is important for monitoring and controlling the temperature of the manufacturing process of semiconductors and ceramics. However, it could be difficult to measure the temperature of moving targets in furnaces or chambers using traditional thermocouples or thermistors because they usually require electric cables for power supply and signal communication. Thus, HT SAW sensing can be a promising technique for these high-temperature applications [64].

**Figure 6.**
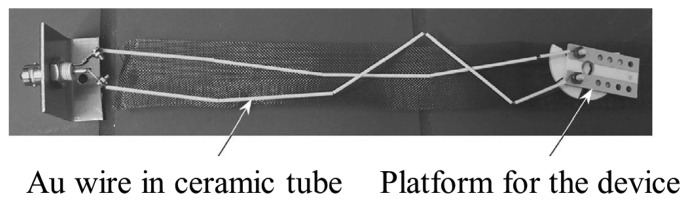
The surface acoustic wave device for high-temperature tests [65] (copyright 2006 by the IEEE).

Hamidon *et al.* have developed a high-temperature surface acoustic wave device using gallium orthophosphate (GaPO_4_) with operating frequency of 434 MHz and temperatures up to 600 °C [65]. Interdigital transducers (IDTs) were fabricated using an e-beam lithography coupled with a lift-off technique on the GaPO_4_ crystal plate. Platinum (Pt) was used for the electrode due to its high melting temperature (1,768.3 °C), high resistance to oxidation and almost constant bulk resistance temperature coefficient. Titanium (Ti) or zirconium (Zr) was chosen as an adhesion layer. The sensor was bonded using Pt wire and attached to a stainless steel substrate for high-temperature tests as can be seen in Figure 6.

**Figure 7.**
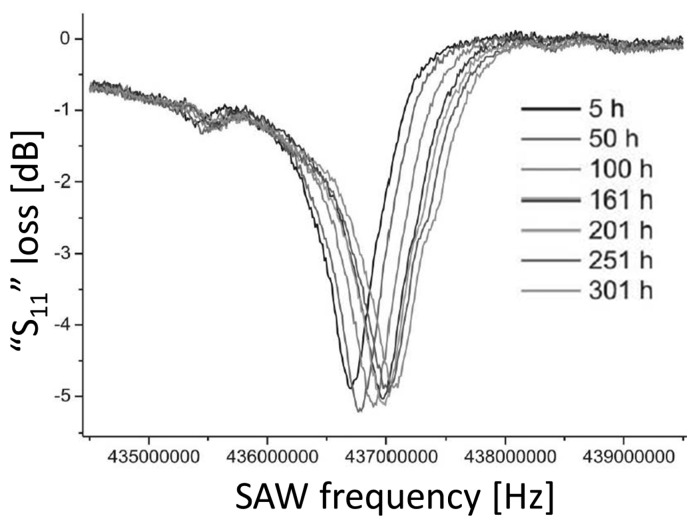
The S_11_ parameters measurement results as a function of frequency at 600 °C for 301 h [65] (copyright 2006 by the IEEE).

The sensor performance and long-term stability was investigated. The S_11_ parameters which indicates the sensor performance was measured as a function of frequency at 600 °C for 301 h. The stable S_11_ loss curve with a frequency shift in a positive direction was obtained as shown in Figure 7. The oxidation at 600 °C was not observed. The prototyped sensor showed the stable performance for more than 3,500 h with high quality factor (3,000–3,500). The results support the use of the developed device as a wireless SAW sensor for high-temperature applications up to 600 °C.

**Figure 8.**
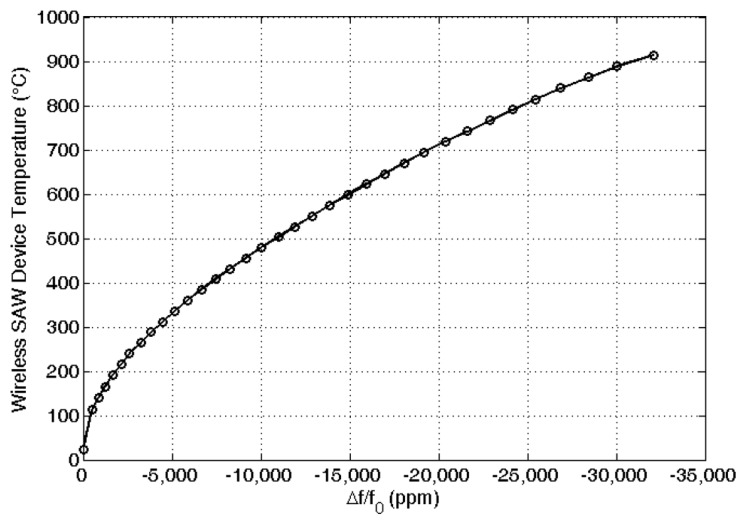
The measured frequency shift with increasing temperatures up to 925 °C [66] (copyright 2011 by the IEEE).

Da Cunha *et al.* have investigated a wireless interrogation of surface acoustic wave (SAW) temperature sensors for harsh-environment sensing applications [66]. SAW sensors were fabricated using lift-off photolithography techniques with langasite (LGS) crystals and thin film deposition of the high-temperature Pt/Rh/ZrO_2_ thin film electrode (thickness: 70–190 nm). To verify the performance of wireless temperature sensors, multiple LGS SAW sensors were wirelessly interrogated in furnace environment and the frequency response of sensors was measured at temperatures up to 925 °C as shown in Figure 8 [66,67].

**Figure 9.**
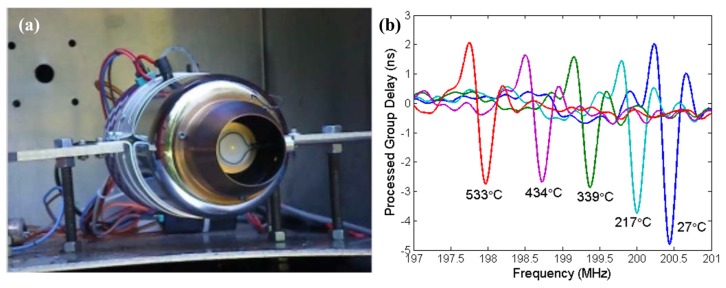
(**a**) SAW sensors mounted on rotating disc of the IBR; (**b**) real-time temperature measurement results during the JetCat operation [66] (copyright 2011 by the IEEE).

In addition, SAW sensors were also tested using the integrally bladed rotor (IBR) of a JetCat turbine engine with temperature up to 750 °C and centripetal acceleration up to 53,000 *g*'s. Wireless SAW sensors were interrogated on the IBR using inductive coupling antennas (Figure 9a) and the temperature-frequency response of SAW sensors was successfully recorded for temperature monitoring of the rotating part (Figure 9b). These results suggest a great diversity of the use of HT SAW wireless sensor for harsh-environmental applications such as aerospace, energy industries and other harsh-environment industrial processes.

**Figure 10.**
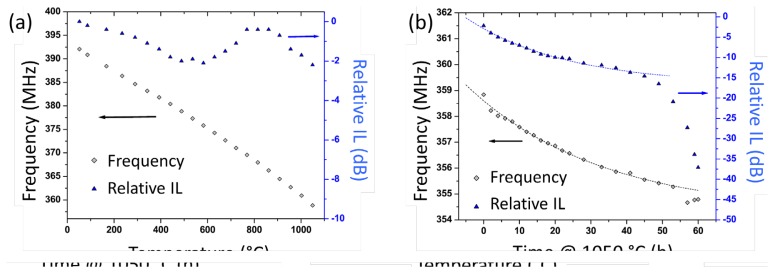
(**a**) The measured frequency shift and relative insertion loss as a function of temperature up to 1,000 °C. (**b**) The measured frequency shift and relative insertion loss as a function time at 1,050 °C [68] (copyright 2013 by the American Institute of Physics).

Aubert *et al.* studied a surface acoustic wave delay lines using AlN/sapphire structure and iridium interdigital transducers for high-temperature applications up to 1,050 °C [68]. The sputtering method was used to fabricate AlN thin films on sapphire substrates. Interdigital transducers were fabricated using iridium (Ir) films as electrodes and titanium (Ti) as an adhesion layer by e-beam evaporation. Photolithography and ion beam etching were used to obtain SAW delay lines. For the high-temperature tests setup, SAW devices were bonded with Pt wires to platinum foil strips and placed inside the furnace. The fabricated SAW sensor was characterized at temperatures up to 1,050 °C under vacuum conditions. The sensor exhibited a large and quasi-linear sensitivity to the tested temperature with a fairly constant insertion loss (IL) as shown in Figure 10a. During the long-term stability test, a significant oxidation of the AlN film was observed, which was a reason of increased IL and the frequency drift after 60 h at 1,050 °C, as can be seen in Figure 10b. Nevertheless, the test results suggest a great potential of AlN-based SAW devices for high-temperature applications at temperatures up to 1,000 °C.

#### 3.3.3. HT PE Ultrasound Transducer

The ultrasonic technique is a popular method to evaluate the internal state of materials, and has been widely used in both laboratories and industrial environments [69]. High-temperature ultrasound transducers (HTUTs) are used for non-destructive testing (NDT) and non-destructive evaluation (NDE) in high-temperature environments [70]. Materials' abnormal behaviors such as corrosion, erosion, defect and wetting behaviors can be evaluated, which usually occur at high-temperatures [71]. HTUTs are also used for the monitoring of advanced composites and engineering materials processing at elevated temperatures, for instance, polymer melting, curing, polymeric foaming and thermoset processing [72–74]. The velocity measurement of hot melt flows using the ultrasound Doppler method is an important field of HTUT applications [75]. The major challenge in HT ultrasonic techniques is to develop transducers which can be operated with stability at ultra-high-temperatures (400–1,000 °C) and can also offer a suitable acoustic coupling between the transducer and the target material [4]. For ultrasonic testing, a number of non-contacting technologies have been developed including electromagnetic-acoustic transducers (EMATs) [76,77], laser generation and detection of ultrasound [78,79], and electrostatic transducers [80]. However, the use of these techniques can be limited by complex process, high testing costs, and low inspection resolution [81]. On the other hand, high-temperature piezoelectric ultrasound transducers (HT-PEUTs) are particularly attractive due to their simplicity, cost-effectiveness and high resolution [70,81,82]. HT-PEUTs can be directly attached to the target at high-temperatures for a short time measurement or a long period during *in situ* monitoring [81].

**Figure 11.**
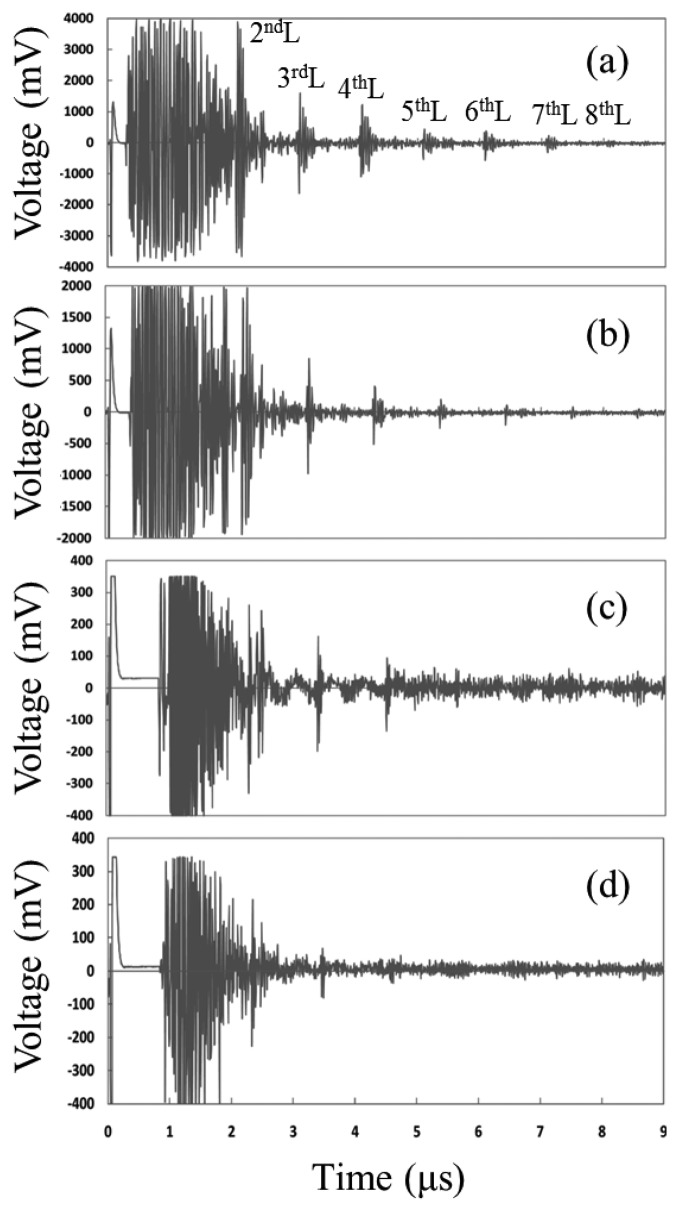
High-temperature pulse-echo test results using AlN thin film ultrasonic transducers at (**a**) room temperature; (**b**) 300 °C; (**c**) 500 °C; (**d**) 550 °C [83] (copyright 2012 by the American Institute of Physics).

Hou *et al.* have demonstrated AlN thin film ultrasonic transducers for high-temperature NDT applications [83]. RF sputter deposition was carried out to fabricate c-axis oriented AlN films on both aluminum alloy and carbon steel substrates. A high-temperature carbon paste and sputtered aluminum were applied to AlN film samples as a back electrode for the electrical connection in high-temperature experiments. High-temperature pulse-echo test results were shown in Figure 11. Multiple echoes were observed even at 550 °C, but the signal strength was significantly decreased. It was revealed that the deterioration of substrate properties and the electrode degradation due to melting, softening and/or surface oxidation were major factors to limit the transducer performance at temperatures higher than 500 °C.

**Figure 12.**
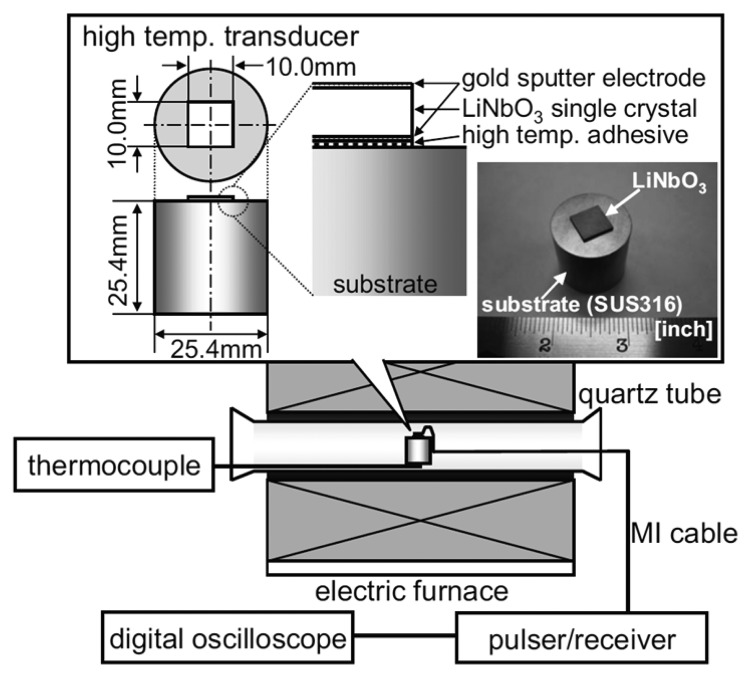
Schematic diagram of an experimental setup for high-temperature ultrasound transducer [84] (copyright 2010 by the American Institute of Physics).

**Figure 13.**
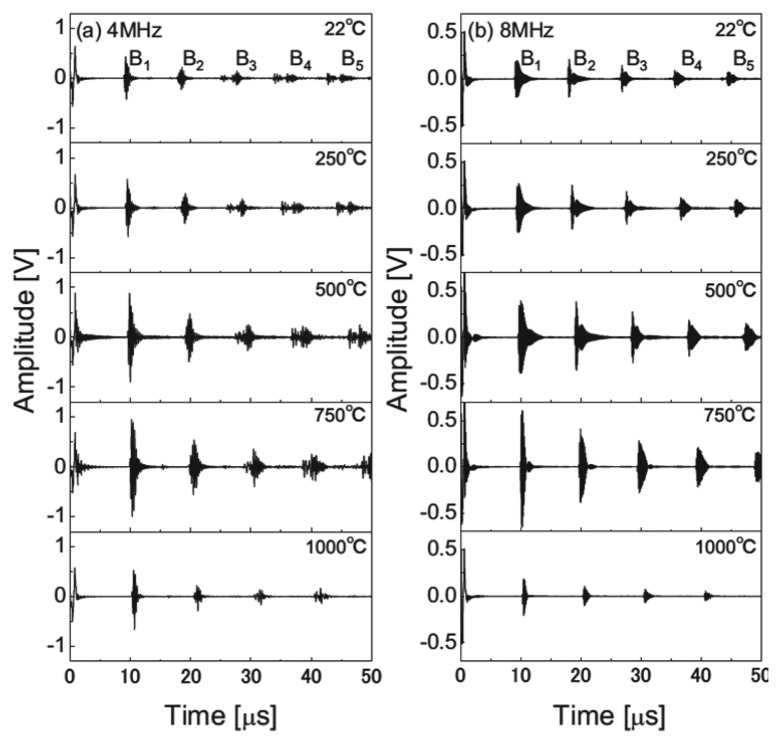
Measured waveforms of multiple echoes at temperatures up to 1,000 °C for (**a**) the 4 MHz transducer and (**b**) the 8 MHz transducer [84] (copyright 2010 by the American Institute of Physics).

Bada *et al.* have developed a high-temperature ultrasound transducer using lithium niobate (LiNbO_3_) single crystals [84]. A stainless steel was chosen as a substrate due to its similar thermal expansion coefficient to LiNbO_3_. The 8 MHz and 4 MHz crystal elements were prepared and bonded onto the substrate using high-temperature adhesive as shown in Figure 12. A high-temperature mineral insulated (MI) cable and silver paste were used for the electrical connection. Allowable temperature limits of all materials were higher than 1,000 °C except for the silver paste. The pulse-echo tests were conducted with the fabricated 8 MHz and 4 MHz ultrasound transducers in a furnace at temperatures up to 1,000 °C. For both transducers the stable multiple echoes were observed from the stainless steel substrate at tested temperatures as can be seen in Figure 13. The transducer failure due to the oxygen loss or the resistance change was not observed during the high-temperature experiments. The test results show that the LiNbO_3_ single crystals can be used for high-temperature ultrasound transducer up to 1,000 °C. These high-temperature results differ from previously reported limitations of 600 °C. This may be due to the temperature dependence of crystal's resistivity [22]. The resistivity of the LiNbO_3_ crystal decreases as operating temperature increases. Decreased resistivity leads to the increased lower limiting frequency (*f*_LL_) of the transducer, since *f*_LL_ is inversely proportional to the *RC* time constant (*i.e.*, $f_{LL}=\frac{1}{2\pi RC}=\frac{1}{2\pi {ɛ}_{0}K\rho }$, where *K* is dielectric constant and *ρ* is resistivity) [21]. Thus, this low resistivity can limit the use of LiNbO_3_ crystal at high-temperature (>600 °C) as a low frequency device, but has minimal impact on high frequency devices.

**Figure 14.**
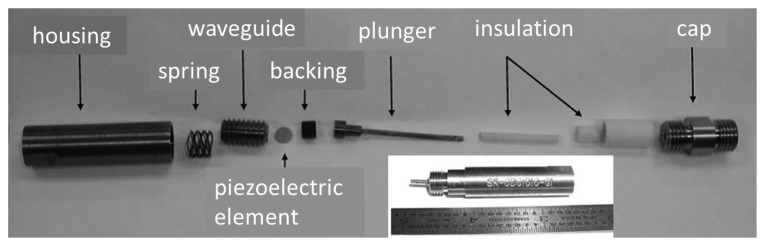
Photograph of the ultrasound transducer and fixture assembly [85] (copyright 2013 by the IEEE).

**Figure 15.**
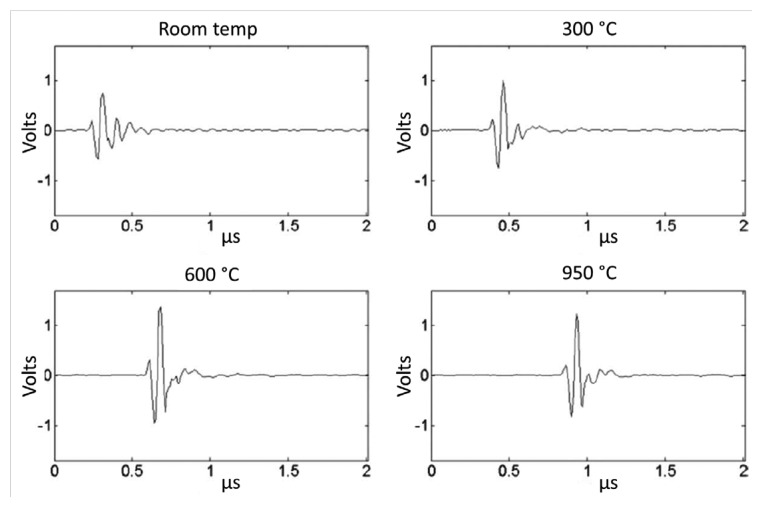
Waveforms obtained from *in situ* test at temperatures up to 950 °C for the YCOB transducer [85] (copyright 2013 by the IEEE).

Recently, Parks *et al.* have studied three different high-temperature piezoelectric materials including YCa_4_O(BO_3_)_3_, LiNbO_3_, and AlN for HT ultrasound transducer applications [85]. Figure 14 shows a photograph of the ultrasound transducer and fixture assembly. A wave spring was used to compress the piezoelectric material onto the ultrasonic propagation medium. Good repeatability (20 times) and longevity (several days) at temperatures below 560 °C were observed with 150 psi of pressure provided by the spring. The echo amplitude was recorded in the long-term *in situ* test for 55 h at 550 °C. It was found that the smaller variation was observed from AlN and YCOB transducers, compared to lithium niobate transducers. It was also revealed that the use of Al foil as a backing material instead of carbon-carbon can improve the transducer performance without the loss of mass or volume at elevated temperatures. In thermal ratcheting tests, each transducer's pulse—echo performance was evaluated with the heat treatments at 950 °C for 24 h and 1,000 °C for 48 h. All three materials exhibited stability in ultrasonic performance but only the YCOB crystal exhibited a much less pronounced change in dielectric properties after heat treatment. The YCOB transducer (using silver foil as the couplant) also showed the stable pulse-echo response from the *in situ* test at temperatures up to 950 °C as shown in Figure 15, which indicates that the YCOB crystals can be promising materials for high-temperature ultrasound transducer applications.

#### 3.3.4. HT PE Acoustic Emission Sensor

Acoustic emissions (AE) are transient elastic waves (100 kHz–1 MHz) which are spontaneously generated by dynamic stress in a structure. As cracks occur or grow in a material body, the elastic energy is released and AE is produced as a result of this failure mechanism. AE sensing techniques using AE sensors are one of the most popular non-destructive inspection techniques [86]. Acoustic emission sensors are usually attached to the surface of testing materials and used to detect the change in the amplitude of elastic waves induced by growing cracks or mechanical failure in structures [87]. From the data obtained using AE sensors, the condition of the target material and the defect location can be monitored continuously. Piezoelectric AE sensors are widely used for various applications due to their low profile. Low profile PE AE sensors can be mounted on the surface of the structure or embedded within the structure directly, improving their response and resistance to environmental effects [86]. High-temperature non-destructive inspection techniques are frequently required for particular applications such as the nuclear power industry. In nuclear power plants, it is important to detect any leaks in valves and pipes as well as the presence of intergranular stress corrosion cracking (IGSCC) [88–90]. The IGSCC problem, for example, is a serious safety issue but also entails the restoration cost which can be in the billions of dollars [91]. Thus, crack growth should be detected instantaneously using a highly sensitive leak and crack detection technique in order to prevent any through-wall cracks in the system at high-temperatures [91]. AE offers promising benefits for this purpose since AE method is an integral method permitting continuous, on-line, and real time monitoring method [92]. For these reasons, piezoelectric acoustic emission sensing techniques for high-temperature applications without using cooling systems or buffer rods have been intensively researched [87].

Kirk *et al.* have reported a piezoelectric acoustic emission sensor using 1–3 piezocomposites for high-temperature applications up to 400 °C [93]. The 1–3 composite was made of lithium niobate crystals (LiNbO_3_) with different crystal orientations (y/36°-cut and z-cut) and different thicknesses (2 mm and 4 mm). High-temperature resistant cements were used as a passive material. For acoustic emission tests, a steel test block and a screen-printed conductive electrode on an alumina substrate were used as electrical connection. An acoustic emission signal was generated by the Hsu-Nielsen method, which has similar acoustic emission properties with that induced by crack growth. For high-temperature acoustic emission tests, a hot plate was used to heat the test block up to 400 °C. Generated acoustic emission signals were successfully detected using prototyped acoustic emission sensors, showing a broad bandwidth and a good signal-to-noise ratio but with some variation in sensitivity due to the low temperature limit of the HT couplant. From this work, it was revealed that prototyped acoustic emission sensors have the ability to detect AE signals up to 400 °C.

**Figure 16.**
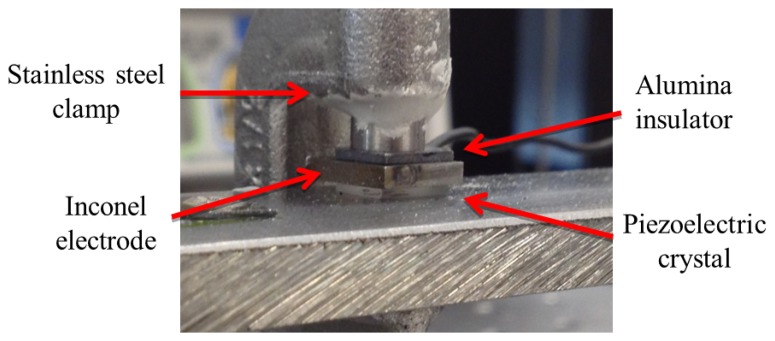
The assembly of the YCOB acoustic emission sensor for HT applications [94] (copyright 2013 by the SPIE).

**Figure 17.**
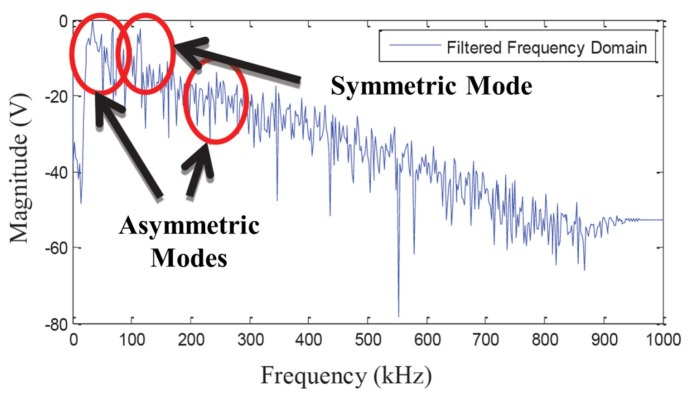
The voltage output of the YCOB acoustic emission sensor as a function of frequency at 1,000 °C [94] (copyright^©^ 2013 by the SPIE).

Recently, Johnson *et al.* have investigated a piezoelectric acoustic emission sensor using YCOB single crystal for use in high-temperature applications [94]. A YCOB crystal was in contact with a stainless steel bar and clamped with an alumina insulator pad using a stainless steel clamp as shown in Figure 16. An Inconel plate was used for the top electrode and Inconel wires were used for electrical connection of the sensor. The sensor was placed inside of a horizontal tube furnace and tested at temperatures up to 1,000 °C. An acoustic emission induced by a Hsu-Nielsen method propagated through the stainless steel bar into a furnace where the sensor was placed. Figure 17 shows the voltage output of the YCOB sensor as a function of frequency at 1,000 °C. It was found that the fabricated acoustic emission sensor was able to detect zero order lamb wave modes and the first order anti-symmetric wave mode successfully at temperatures up to 1,000 °C. No degradation of the sensor performance was observed during the high-temperature tests. The results of this study support the feasibility of YCOB single crystals as acoustic emission sensor materials for high-temperature applications.

#### 3.3.5. HT PE Gas Sensor

**Figure 18.**
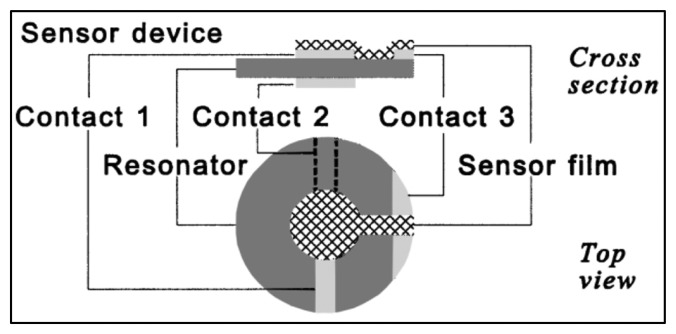
Schematic of a nanobalance gas sensor with selective film [30] (copyright 2001 by the Elsevier Science Ltd).

Gas sensors present value in many applications, especially high-temperature combustion processes. Piezoelectric gas sensors can be created by means of microbalances with deposited layers of film that selectively absorbs gas molecules. Shifts in the resonant frequency of the device are accompanied by the changes in mass, which can be quantitatively related. Quartz crystal microbalances (QCMs) are commonly used due to their low cost, but are limited to the operating temperature of quartz, ∼350 °C. Research on developing a high-temperature gas sensor using this structure has been conducted, using a langasite LGS crystal and TiO_2_ film for an oxygen selective gas sensor [30,95]. The sensor schematic is shown in Figure 18, with references to the 780 μm thick LGS resonator, platinum electrodes, and the O_2_ sensitive TiO_2_ film.

**Figure 19.**
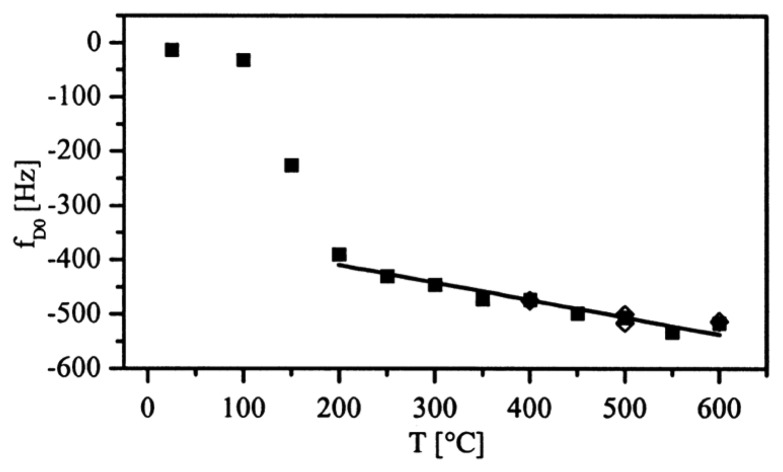
Frequency drop (*f*_D0_) of the prototyped LGS gas sensor with increasing temperatures and different atmospheric conditions (O_2_ to 6% H_2_/Ar) [30] (copyright 2001 by the Elsevier Science Ltd).

Figure 19 shows the frequency drop (*f*_D0_) of TiO_2_ film coated LGS gas sensor resulted from the atmospheric condition change (O_2_ to 6% H_2_/Ar) with increasing temperatures. It was observed that the prototype nanobalance gas sensor was able to detect O_2_ gas effectively at temperatures up to at least 600 °C. The temperature dependence of the sensor was accounted for, and reference nanobalance sensors were used to reduce the temperature sensitivity [30].

Similar research for high-temperature gas sensors has been conducted by Zheng *et al.* [96]. High-temperature oxygen gas sensor was fabricated using LGS resonators with ZnO sensing films and the O_2_ response of the sensor was successfully tested at temperatures ranging from 500 °C to 700 °C. These results revealed that LGS resonators can be reasonably used as high-temperature gas sensors with a proper gas selective sensing film for different gases detections.

#### 3.3.6. HT PE Pressure Sensor

**Figure 20.**
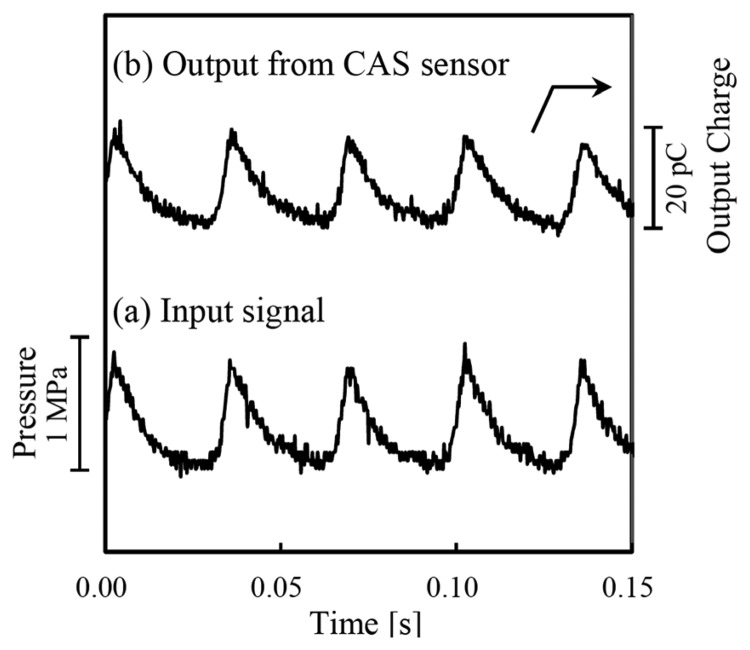
Output from CAS high-temperature pressure sensor at 700 °C [97] (copyright 2013 by the American Institute of Physics).

High-temperature piezoelectric pressure sensors have been developed in both industry and in academic research institutes due to their criticality in combustion systems. Pressure is a common measurement used in internal combustion engines and turbines, where pressure, temperature, and flow rates are necessary units of measurement for control. A high-temperature piezoelectric pressure has recently been developed by Takeda *et al.* by using calcium aluminate silicate (CAS) single crystals (CAS crystal belongs to the melilite family) [97]. The crystals were grown using the Czochralski method and were tested in bulk crystal form. The crystals produced promising sensitivity at high-temperatures, although it was not quantitatively measured above room temperature. The output from a stress wave is shown in Figure 20, which was measured at 700 °C.

High-temperature pressure sensors have likewise been developed by companies such as Kistler, who offers a sensor using their PiezoStar crystal, which is similar in structure to langasite [98]. In their work, a high-temperature pressure sensor was designed, fabricated and tested. The prototyped pressure sensor consisted of PiezoStar crystal KI100 working in transverse mode, nickel based alloys for base materials and mineral insulated cables for electrical connections. Their sensors have been tested for long-term high-temperature exposure by ageing them at 600 °C for up to 2,000 h. The high-temperature aging tests results showed the prototyped sensor's reliable stability for long-duration use with the small deviation (−2% to +4%) in sensitivity (45 pC/bar). The deviation that did occur is most likely due to property changes in the sensor structure, not the crystal itself.

## 4. Conclusions and Future Work

High-temperature sensors play a significant role in the aerospace, aircraft, automotive and energy industries for achieving improved fuel efficiency, reduced emissions and decreased maintenance cycles and failures. High-temperature piezoelectric sensing is one of the most promising techniques due to its high-temperature stability and reliability, simple and lightweight structural design as well as high cost effectiveness. Langasite (LGS), LiNbO_3_ (LN), AlN and YCa_4_O(BO_3_)_3_ (YCOB) are the most widely used piezoelectric materials for ultrahigh-temperature applications due to their stable performance at elevated temperatures. HT YCOB devices in particular, including accelerometers, ultrasound transducers, and acoustic emission sensors show a highly steady and reliable operation even at temperatures over 1,000 °C. However, some challenges are ahead as well: (1) the crystal property problem: changes in dielectric properties can be troublesome, but decreased resistivity at elevated temperature is the most critical problem. Suitable PE materials should be chosen considering the relationship between the resistance and the lower limiting frequency of the sensor; (2) the electrode problem: electrode materials such as Pt undergo degradation at high-temperature (>1,000 °C) which can leads to device failure. Electrodeless devices could be one option, but stable performance is difficult to achieve, especially in long-term experiments. The use of conductive oxide ceramic materials could be a solution for this issue; (3) the electronic connection and bonding problem: besides PE and electrode materials, other electronic components such as electric wires and conductive bonding for high-temperature operation likewise bring forth critical issues. Mineral insulated (MI) cable can be a good option for high-temperature use less than 1,100 °C. Inconel wires shows a good conductivity at high-temperature (>1,000 °C) but the noise induced from the environment needs to be reduced. Bonding materials such as epoxy are usually not suitable for high-temperature due to their low melting point. Bolt/nut assembly without bonding present a possible solution, compensating the thermal expansion problem; (4) the sensor package problem: in harsh environments, it is important to create a package to reduce the high-temperature exposure for the sensor components and to reduce the electrical and mechanical noise. The use of protective coating on the sensor device is one option; (5) backing material for HTUT: backing material is a crucial problem for high-temperature ultrasound transducers due to noises caused by imperfect damping in backing materials at HT. Recent work reveals that carbon-carbon material shows good performance as a backing material at high-temperatures (>500 °C) for long-term use (>55 h) [85].

## Figures and Tables

**Table 1.** Key features of existing high-temperature sensors [7,12,16,17,19–21].

**Features**	**Piezoresistive**	**Capacitive**	**Piezoelectric**	**Fiber Optic**
Measured parameters	Resistance	Capacitance	Charge and voltage	Intensity and phase
Resolution (strain)	10^−6^	10^−6^	10^−12^	10^−1^
Frequency range	DC -kHz	DC- kHz	1 kHz- MHz	DC- 100 kHz
Temperature range (°C)	<600	<400	<1,000	<1,100
Temperature stability	Poor	Good	Good	Good
Integration	Easy	Easy	Easy	Hard
Power consumption	Medium	Medium	Low	High
Cost & complexity	Low	Low	Low	High
Electrode degradation	No	Yes	Yes	No

**Table 2.** High-temperature piezoelectric materials [3,21,36–39].

**Material**	**GaPO_4_**	**LN**	**LGS**	**YCOB**	**GdCOB**
T_max_ (°C) (phase transition or melt)	970	1150	1470	1510	1470
T_use_ (°C) (suggested usage temp) #	700	600	800	∼1,250	<1,200
Temperature limited by	Phase transition & attenuation	Resistivity	Resistivity	Resistivity	Resistivity
Mechanical quality factor †	10,000	2,000	15,000	9,000	5,000
Measured temperature (°C)	800	500	600–500	1,000–800	1,000–800
Dielectric loss	0.15	5.5	0.5–0.2	0.3–0.1	0.3–0.2
Dielectric permittivity variation (%)	5	∼40	25–15	15–10	10–9
Resistivity (Ω)·cm;	2.3 × 10^7^	6.6 × 10^5^	2 × 10^6^–8 × 10^6^	1 × 10^7^–2 × 10^8^	4 × 10^6^–3 × 10^7^

# The suggested usage temperatures are based on a standard of 1 MΩ).cm resistivity for comparison purpose (with the exception of LN;, materials with lower resistivity may still be functional in different applications, for example, LN crystals can be used up to 1,000 °C for short term and high frequency range [21].

† The mechanical quality factors were at room temperature, closely related to various vibration modes, the values reported for ReCOB crystals are the thickness shear mode [21].

**Table 3.** Thin film electrodes (∼100 nm) for high-temperature applications [40–44,46–52].

**Thin Films**	**Operating Temperature (°C)**	**Advantages/Disadvantages**	**Adhesion Layer**	**Applications**
Pt	650–750	Surface degradation at temperatures below 600 °C	N/A	Piezoelectric Sensors
Ir/TiAlN	700	Better O_2_ diffusion barrier than that of Pt electrode	TiAlN	Ferroelectric capacitors
Pt/Ir, Pt/Rh	750	Excellent fatigue resistance and stable up to 750 °C	N/A	Ferroelectric capacitors
Pt/Zr	750	No metal surface degradation up to 750 °C	Zr	SAW devices
Pt-10%Rh/ZrO_2_	850	Film recrystallization, agglomeration, and dewetting (700–1,000 °C)	Zr	SAW devices
LSCF and LSM	800	Stable up to temperatures of 800 °C; good conductivity at temperatures above 600 °C	N/A	Solid oxide fuel cell, SAW sensors
